# Immune System Stimulation by Oncolytic Rodent Protoparvoviruses

**DOI:** 10.3390/v11050415

**Published:** 2019-05-04

**Authors:** Assia Angelova, Jean Rommelaere

**Affiliations:** German Cancer Research Center (DKFZ), 69120 Heidelberg, Germany; a.angelova@dkfz-heidelberg.de

**Keywords:** rodent protoparvoviruses, oncolytic activity, tumor microenvironment, immunomodulation, preclinical, clinical trials

## Abstract

Rodent protoparvoviruses (PVs), parvovirus H-1 (H-1PV) in particular, are naturally endowed with oncolytic properties. While being historically described as agents that selectively replicate in and kill cancer cells, recent yet growing evidence demonstrates that these viruses are able to reverse tumor-driven immune suppression through induction of immunogenic tumor cell death, and the establishment of antitumorigenic, proinflammatory milieu within the tumor microenvironment. This review summarizes the most important preclinical proofs of the interplay and the cooperation between PVs and the host immune system. The molecular mechanisms of PV-induced immunostimulation are also discussed. Furthermore, initial encouraging in-human observations from clinical trials and compassionate virus uses are presented, and speak in favor of further H-1PV clinical development as partner drug in combined immunotherapeutic protocols.

## 1. Bystander Antitumor Effect of Protoparvovirus-Induced Oncolysis

As reviewed recently [1,2], rodent protoparvoviruses are endowed with oncolytic properties. The molecular basis of protoparvovirus cancer cell specificity and killing activity is the subject of another review in this special issue [3]. In some cancer animal models, this direct viral oncolytic effect is potent enough to fully eradicate infected tumors, correlating with virus spread and viral oncotoxic protein NS1 expression throughout the neoplastic tissue [4]. In most models, however, intratumoral virus multiplication and propagation are limited. Protoparvovirus-induced tumor suppression can still take place in such systems, where only a minor fraction of tumor cells gets lytically infected [5,6]. An extreme case is the one of animals implanted with double tumors, in which the protoparvovirus-induced lysis of the infected tumor leads to regression of the non-infected distant tumor, in the absence of virus transfer [7,8]. These data speak for the involvement of an immune bystander effect taking over from the initial direct viral oncolytic effect to complete tumor elimination. The tight cooperation between protoparvoviruses and the immune system to synergistically achieve tumor suppression is evidenced by a number of phenomenological observations and mechanistic investigations, as reviewed below. Most of these studies concern the rodent protoparvovirus H-1PV and (tumor) cells of either rat (the natural host of this virus) or human origin. Since H-1PV is not infectious for mouse cells, a few cited works were carried out in murine models with closely related mouse protoparvoviruses, in particular the prototype strain of the minute virus of mice (MVMp). H-1PV and its close relatives will be collectively designated PVs in this review.

## 2. Phenomenological Evidence of PV-Immune System Cooperation

Animals cured of their cancer as a result of PV treatment develop tumor-specific memory responses protecting them against subsequent challenges with the same tumor cells, in the absence of detectable viral imprints [5,9,10,11]. This long-term vaccination effect is somewhat expected from the viral oncolysis-dependent release of tumor-associated antigens (TAAs) triggering tumor-specific adaptive cellular immune responses. Besides protecting animals from cancer recurrence, these responses also contribute to PV-induced elimination of primary tumors, as indicated by the ability of H-1PV to enhance the efficiency of an autologous tumor cell-based therapeutic vaccine [7].

Direct indications of the role of the immune system in PV-mediated tumor destruction were obtained through different complementary approaches.

- PV antineoplastic efficacy is higher in immunocompetent, as compared to immunodeficient animals. The impairment of the acquired cell-mediated arm of the host immune system by genetic means [11,12], or by experimental cell depletion [7,13], was indeed found to correlate with reduced PV capacity for tumor suppression.
- Adoptive transfer of splenocytes from rats undergoing H-1PV-mediated tumor regression into naïve animals bearing the same tumor protects the recipients against cancer development, in absence of detectable virus transmission [14].
- Animals undergoing tumor suppression upon PV treatment show distinct changes in tumors and lymphoid tissues, pointing to the induction of Th1-type cellular immune responses. This induction was revealed through the elevated production of cytokines (notably interferon (IFN)-γ and tumor necrosis factor (TNF)-α), the infiltration of tumors with activated helper and cytotoxic T lymphocytes (CTLs), and the proliferation of cytotoxic and/or helper T cells in spleen and tumor-draining lymph nodes [7,8,12,14,15]. While this response is likely to be directed mostly against viral epitopes, its stimulation by uninfected tumor cells under in vivo and/or in vitro conditions argues for at least some level of tumor specificity [11,16].
- Upon H-1PV infection, human pancreatic carcinoma cells can prime human immune cells to inhibit tumor development. This was shown in a humanized patient-derived xenograft model, using ex vivo primed human dendritic and T cells for immunodeficient mice reconstitution and growth suppression of pancreatic cancer cells derived from the same patient [15].
- There is a first hint of H-1PV oncosuppressive capacity enhancement through co-treatment with immunostimulants. In a model of late (peritoneal carcinomatosis-associated) pancreatic cancer, co-application of IFN-γ improved H-1PV-mediated control of the disease [16]. This improvement correlated with enhanced activability of isolated peritoneal macrophages (TNF-α production) and splenocytes (proliferation).
- PV propensity for inducing Th1 environment is substantiated by the bias of the virus-neutralizing humoral response elicited after infection towards Th1/IFN-γ-dependent IgG2a isotype antibodies [17,18]. Furthermore, some PVs were found to potentiate autoimmune reactions through the modulation of T cell effector functions [19,20].

A straightforward mechanism of PV priming of antitumor immune reactions would be the release of cellular TAAs and/or viral antigens as a result of the lytic infection of tumor cells, leading to induction of tumor-specific responses and the generation of a proimmune milieu. Yet another non-exclusive possibility deserves to be considered. PVs may act on the immune system, either directly, by infecting various immune cells, or indirectly, by causing infected (tumor) cells to produce viral and/or cellular signals (the so-called pathogen- and damage-associated molecular patterns, PAMPs and DAMPs, respectively), which are recognized by immune cells, and regulate their activity. Two pieces of in vivo evidence support the latter possibility.

- In mice infected with MVMp, extratumoral viral gene expression has been detected in lymphoid tissues [18] and assigned to rare subpopulations of cells known to play a role in cancer immune surveillance, namely myeloid dendritic cells (DCs) and B1 lymphocytes [21]. Interestingly, MVMp-infected animals show striking upregulation of the expression of IP-10, a chemoattractant known to be produced by these cells and to have antitumoral properties. In a rat model of pancreatic carcinoma, an initial burst of extratumoral H-1PV expression has also been observed in lymphoid organs [6].
- A first indication of H-1PV intrinsic immunostimulatory activity has been obtained for virus mutants that are endowed with higher anticancer potency, while keeping the same oncolytic efficacy as the wild-type virus. These mutants were obtained by arming the PV genome with known immunostimulating PAMPs, namely unmethylated CpG motifs. The CpG mutants proved superior to the original virus at inducing the above-mentioned immunological changes in tumors and lymphoid tissues, in particular DC activation in tumor-draining lymph nodes [15,22].

## 3. Mechanistic Evidence of PV Capacity for Modulating the Immune System

The ability of PVs to upregulate the immune system has been demonstrated through a number of in vitro studies using immune cell cultures or co-cultures with tumor cells.

PV immunostimulating activity is mediated in part by tumor cell factors whose expression is modulated by virus infection. PVs have been found to kill tumor cells through multiple mechanisms (for reviews, see [1,2,23]). Besides being multimodal, PV-induced tumor cell death has been proved to be immunogenic.

- A first hint of the interconnection between H-1PV and immunogenic cell death (ICD) has been given by the observation that human myeloid leukemia cell variants selected for their resistance to the virus also resisted TNF-α, a known inducer of the release of a plethora of proinflammatory DAMPs and cytokines [24].
- H-1PV infection makes human melanoma cells able to trigger the activation/maturation of innate immune cells, DCs in particular [25]. Similarly, microglia and DC subsets get activated after co-culture with MVMp-infected mouse glioma cells [11]. PVs are much more potent than other inducers of tumor cell death in having this immunostimulating effect. H-1PV-dependent DC activation has been found to correlate with strong and long-lasting release of the DAMP heat shock protein (HSP) 72 by infected human melanoma cells [25]. DCs incubated with H-1PV-induced melanoma cell lysates show increased expression of both specific Toll-like receptors (TLRs) and NF-kB, arguing for a role of TLR signaling in virus-mediated maturation of DCs [26].
- In keeping with their relaying role between innate and adaptive immunity, human DCs activated by H-1PV-induced tumor cell lysates are able to phagocytose these lysates and cross-present TAAs, leading to the stimulation of CTL clones specific for these epitopes [27,28].
- H-1PV infection confers to human pancreatic and colon carcinoma cells an enhanced capacity for stimulating natural killer cells (NKCs) to release cyto/chemokines and kill tumor cells [29,30]. This H-1PV-mediated increase in NKC oncotoxic activity has been traced back to both the overexpression of ligands specific for various NKC activation receptors and the down-modulation of MHC class I molecules on virus-infected tumor cells.
- In agreement with the above data, incubation with H-1PV-infected human pancreatic carcinoma cells induces Th1/M1 immune signature in human peripheral blood mononuclear cells (PBMCs), as revealed in particular by the enhanced production of IFN-γ and TNF-α [14]. These changes are intriguing, given their known association with tumor immune rejection. This modulation was achieved by infected pancreatic cancer cells, which are unable to support virus production, and is therefore likely to result from immunogenic signals produced by infected tumor cells instead of PBMC infection by progeny virions. It is worth noting that H-1PV can exert, in addition, direct effects on PBMCs, as discussed below.

The immunostimulating activity of PV-induced tumor cell lysates can be boosted by co-application of immunomodulators, or by virus manipulation

- H-1PV cooperates with other inducers of ICD, resulting in the production of a broader spectrum of DAMPs by co-treated tumor cells. This can be exemplified by an H-1PV/gemcitabine chemovirotherapeutic treatment, whose capacity for inducing human pancreatic cancer cells to release two main markers of ICD, the DAMPs high mobility group box (HMGB) 1 and ATP, relies on the virus and the drug, respectively [31]. In agreement with these data, H-1PV and gemcitabine act synergistically to induce pancreatic carcinoma cells to activate co-cultured human PBMCs, as revealed by the production of IFN-γ [15].
- Another intriguing strategy for improving the immunostimulating activity of PV-induced tumor cell lysates consists of combinations of immune checkpoint blockers to remove inhibitory signals of T cell activation. First credit to this application was given by the potentiating effect of sunitinib, a receptor tyrosine kinase inhibitor with immune checkpoint blockade properties, on the ability of H-1PV-infected human melanoma cell lysates to induce DC cross-presentation-dependent activation of tumor antigen-specific CTLs [28]. Furthermore, the immune checkpoint-blocking antibody tremelimumab may stimulate human DC maturation mediated by H-1PV-induced colon carcinoma cell lysates [32].
- Arming the H-1PV genome with immunostimulating CpG elements boosts virus capacity for inducing the above-mentioned tumor surveillance-predictive IFN-γ/TNF-α signature, upon infection of co-cultures of human PBMCs and pancreatic cancer cells [15].

Besides inducing tumor cells to produce immunostimulating signals, PVs can also infect distinct immune cells and activate them in a direct way. This appears to be true in spite of the remarkable oncotropism, which restricts the effects of PVs on normal tissues [1]. PVs can indeed enter many normal cells, and while being abortive, infection may still have physiological impacts, particularly on the immune system. Infection of human PBMCs with H-1PV is abortive, leading to no detectable production/release of progeny virions [14,33]. Analysis of H-1PV life-cycle in distinct immune cells shows that virus entry takes place in T, B and NK lymphocyte subpopulations, monocytes and DCs, but replication gets blocked at various subsequent steps, with no or limited production of the viral cytotoxic protein NS1 [14,25,29,33,34]. Human neutrophils also prove to be non-permissive for H-1PV [33]. A similar abortion of MVMp life-cycle has been observed after infection of human PBMCs [34], mouse splenocytes [21], DCs [11] and glial cells [35]. The abortive PV infection of isolated immunocytes causes no or little harm to these cells [25,29,33,35]. However, H-1PV infection of human PBMCs is associated with significant toxic effects for which B lymphocytes or NKCs may be targets [14,33]. This cytotoxicity appears, at least in part, not to be a direct consequence of virus infection, but to be mediated by cellular factors that accumulate in vitro after being released from distinct immune cells as a result of their activation by H-1PV.

- Some normal human immune cells appear to respond to PV infection by producing type I IFNs, as detected in human PBMCs exposed to H-1PV or MVMp [34,36]. Distinct human immune cells, most likely plasmacytoid DCs, appear to sense PV infection through TLRs and possibly also through other receptors [34]. The activation of type I IFN response in these cells may contribute to their resistance to PV infection due to abortion of virus replication (see above). This response may still be host range-dependent, as MVMp failed to induce similar type I IFN production in mouse plasmacytoid DCs [37]. It is noteworthy, however, that some non-immune normal cells may also be induced to produce type I IFNs upon PV infection, depending on the host cell origin. While the capacity of MVMp for triggering type I IFN production has been demonstrated in normal fibroblasts derived from mice, the natural host of this virus [38,39,40], PVs have failed to evoke detectable type I IFN response in a number of normal human cell types [36]. Altogether, these observations indicate that although many human cells may fail to develop type I IFN response after being exposed to rodent PVs, a distinct subset of immune cells are able to sense PV infection and sustain significant type I IFN production. Besides having antiviral functions, type I IFNs exert a wide range of stimulatory activities on both the innate and acquired arm of the immune system. It therefore seems justified to include these cytokines in the series of potential mediators of PV immunostimulation.
- PV infection of immune cells can have other phenotypic impacts besides type I IFN induction. The analysis of the functional impact of H-1PV infection on isolated subsets of human immune cells has revealed virus capacity for activating T helper cells (expression of activation markers and secretion of Th interleukins (ILs)) [33], macrophages (TNF-α release) [14] and DCs (TNF-α and proinflammatory IL production, expression of type I IFN-stimulated genes) [15]. A weak stimulating effect of MVMp infection on mouse DCs has also been reported [11]. Direct PV infection appears to be less efficient at activating DCs than incubation with PV-infected tumor cells [27]. In contrast to the stimulation of the above-mentioned immune cells, a down-regulating effect of H-1PV has been observed for regulatory T cells whose suppressive activity is inhibited by infection [33]. It is worth noting that the immunostimulatory signals induced by direct PV infection overlap those induced by incubation with infected tumor cells (see previous sections), suggesting that some of the activating effects of the latter cells may be mediated by PAMPs, as well as the above-mentioned cellular DAMPs. In agreement with the phenotypic changes induced in individual immune cells, H-1PV infection of PBMCs generates a TNF-α/IFN-γ/IL-2 signature that is accompanied by activation and focal proliferation of T cells, with the prevalence of CD4^+^ Th cells [14,15,33]. Similarily, conditioned immunocytes from mouse spleen and lymph nodes sustain enhanced IFN-γ production after MVMp infection [21]. Altogether, these observations are indicative of PV direct capacity for Th1-biased immune upregulation. In agreement with the above-mentioned TLR involvement in PV induction of type I IFN production by PBMCs, an H-1PV mutant armed with CpG motifs proves to be more effective than the wild-type virus in triggering antigen-presenting and T cell activation, and IFN-γ, IL-2 and type I IFN release after infection of human PBMCs [15].

## 4. Conclusions: Use of H-1PV and Its Relatives to Fine-Tune Immune Responses

**Figure 1:**
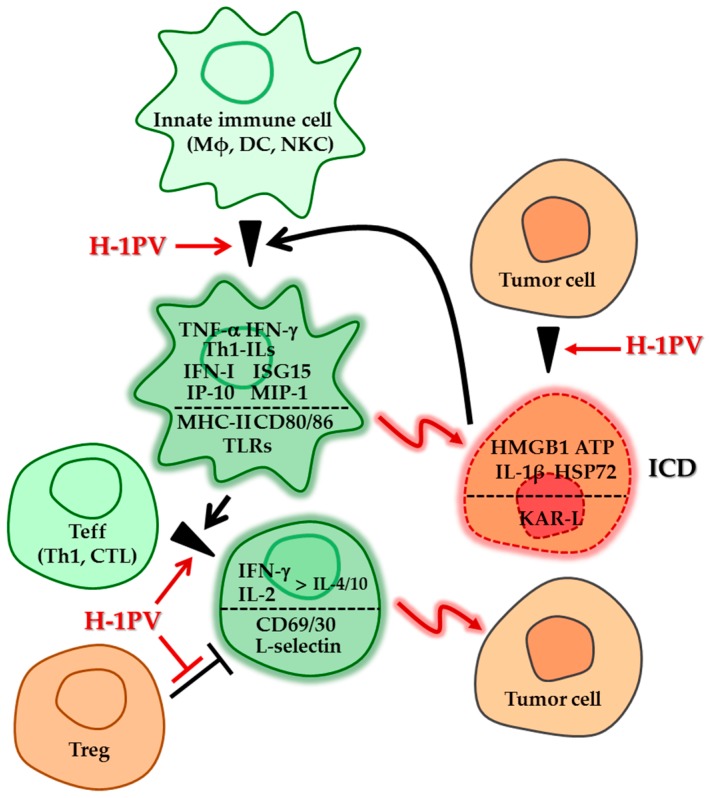
Preclinical evidence of H-1PV impact on the crosstalk between immune and tumor cells. Innate and adaptive immune cells are stimulated as a result of both their contact with H-1PV-infected tumor cells and their direct infection with the virus. This immunostimulatory effect of H-1PV is revealed through the induction of markers of immunogenic death in infected tumor cells and of phenotypic activation in immune cells. The mediators involved include a number of cytokines/extracellular signaling molecules and cell membrane receptors/ligands, as listed for the corresponding cell types (above and below the dotted line, respectively). For details and references, see main text. ATP, adenosine triphosphate; CD, cluster of differentiation; CTL, cytotoxic T lymphocyte; DC, dendritic cell; HMGB, high mobility group box; HSP, heat shock protein; ICD, immunogenic cell death; IFN, interferon; IL, interleukin; IP, interferon-gamma-induced protein; ISG, interferon-stimulated gene; KAR-L, killer activation receptor ligand; Mφ, macrophage; MHC, major histocompatibility complex; MIP, macrophage inflammatory protein; NKC, natural killer cell; Teff, effector T cell; Th, T helper; TLR, Toll-like receptor; Treg, regulatory T cell.

The above data give credit to the ability of PVs to (in) directly interact with the immune system and generate a microenvironment favorable to the development of both innate and acquired cell-mediated immune responses (Figure 1). While resulting in part from phenotypic changes directly induced by PVs in immunocytes, immune upregulation is exacerbated in the presence of tumors. This tumor dependence reflects the fact that infected neoplastic cells are factories for the production, not only of progeny virions, but also of PAMPs and DAMPs, which act to alert the immune system. In consequence, PV-mediated immunostimulation proves to be directed, at least in part, against tumors in various model systems. The potential application of this property to cancer therapy raises the question of whether the immunomodulating activity of PVs poses any risk to the host. Two lines of evidence speak for H-1PV and MVMp being friendly immunostimulators.

- The PV-mediated immunological activation observed in in vitro models takes place in the absence of major toxicity for immune cells, which undergo an abortive infection with no or few direct cytopathic effects (see above). It should, however, be stated that this conclusion cannot be extended to all rodent PVs, some of which target cells of the hematopoietic system and can lead to immune dysfunctions [41].
- Animal studies show that infection of natural hosts with H-1PV and MVMp, even at very high doses and repeated treatment, is not associated with any immunotoxicity or threatening overactive immune responses, such as cytokine storms, autoimmunity or overt inflammation [18,42]. Furthermore, PV adjuvant effects described above in cancer animal models were not accompanied by any harmful immunological side effects [6,12]. Therefore, these viruses have a generally low proinflammatory profile and depend on the presence of neoplastic tissues to exert immune adjuvant effects that are targeted at infected tumors and surrounding lymphoid organs. This tumor specificity of danger signaling by H-1PV and MVMp speaks for the inclusion of PV-based treatments in the developing arsenal of cancer immunotherapies.

## 5. First Clinical Hints of H-1PV Capacity for Tumor Microenvironment Immunomodulation in Cancer Patients

The above described preclinical evidence of H-1PV capacity to exert immunostimulatory effects in various cancer models raises the question of whether similar observations could also be made in a clinical context, i.e., in H-1PV-treated cancer patients. In 2011, the first-in-man PV clinical trial (ParvOryx01) was launched in recurrent glioblastoma patients [43]. Trial initiation was prompted by preclinical reports of Geletneky et al., demonstrating striking PV-induced tumor regression in intratumorally and systemically treated glioma-bearing animals [10]. First clinical experience brought much essential knowledge, which laid the ground for further H-1PV clinical developments. ParvOryx01 demonstrated that the virus exhibits a reliable safety and tolerability profile in both local and systemic applications, and poses no risk of environmental contamination or undesired transmission to third persons. Furthermore, intratumoral H-1PV expression was also documented in virus local injection site-distant tumor areas, and in intravenously treated patients. H-1PV capacity to cross the blood-brain/tumor barrier, already described in animal glioma models [10], was therefore confirmed in men. The progression-free and overall survival of ParvOryx01 patients compared favorably with published meta-analyses of recurrent glioblastoma cases [44]. Notably, ParvOryx01 provided observations in support of H-1PV’s double-faceted mode of action as both an oncolytic and immunostimulatory anticancer agent. Virus-specific T cells were detected in the peripheral blood of the majority of ParvOryx01 patients [44]. Although antiviral immune responses are generally considered restrictive for the efficacy of OV therapy, growing evidence suggests that they can reverse the tumor-driven host immune suppression by inducing ICD, facilitating the initial priming of antitumor immune responses, and establishing a niche suitable for the development of tumor-specific immunity [45]. It is indeed noteworthy that glioma-specific peripheral T cell responses were detected in half of the tested H-1PV-treated glioblastoma patients [44].

**Figure 2:**
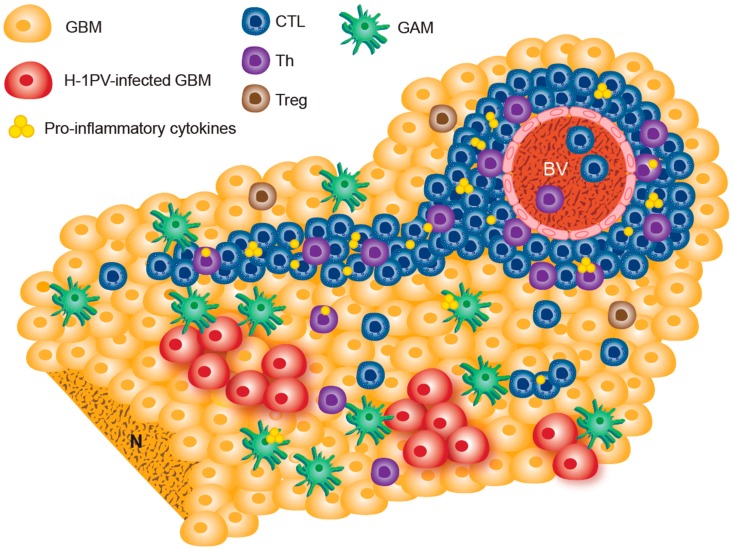
Tumor microenvironment immune landscape as identified in resected tumors from H-1PV-treated recurrent glioblastoma patients. Microglia/macrophage activation, diffuse and perivascular tumor infiltration with activated Th and cytotoxic T cells, and proinflammatory cytokine expression were observed in patient-derived tumor tissue sections. Only scarce Treg cells were present. H-1PV transcripts as well as the oncotoxic NS1 protein were detected in clustered tumor cells. For details and references, see [44,46] and main text. BV, blood vessel; CTL, cytotoxic T lymphocyte; GAM, glioblastoma-associated microglia/macrophages; GBM, glioblastoma; N, necrosis; Th, T helper; Treg, regulatory T cell.

The availability of resected glioblastoma tissues allowed the analysis of the tumor microenvironment (TME) nine days after H-1PV treatment administration. In comparison with historical controls, in patients who received H-1PV treatment, activated granzyme B and perforin-positive CTLs and Th cells massively infiltrated the tumor. Both perivascular and diffuse intratumoral immune infiltrates were observed [44,46]. In contrast, only scarce, single scattered Treg cells were seen. IFN-γ and IL-2 expression was also detected in these tumors. Glioblastoma-associated microglia/macrophages (GAM) displayed pronounced CD68 and cathepsin B (CTSB) upregulation, characteristic of an activated state (Figure 2). Of note, apoptosis of glioma cells induced by microglia-derived secreted CTSB has been shown in vitro [47].

The above data hint at the establishment in H-1PV-treated glioblastoma patients of a “hot”, proinflammatory TME, which may facilitate tumor targeting by host antitumor immune responses. This offers the possibility to synergistically increase TME “warming up” by combining H-1PV with other immunotherapeutic strategies. Indeed, H-1PV was recently combined with bevacizumab [48] or bevacizumab and checkpoint inhibition [49,50] within the frame of a compassionate virus use program. This H-1PV-based viro-immunotherapeutic approach achieved high rates of objective antitumor responses in glioblastoma patients, raising increased expectations towards the efficiency of the concept. It is noteworthy that bevacizumab, although originally developed as an antiangiogenic drug, exerts in addition a certain degree of immunomodulation. Bevacizumab reduces vascular endothelial growth factor (VEGF)-induced defects in DC functions, and inhibits tumor infiltration by immune regulatory cells, such as Treg and myeloid-derived suppressor cells (MDSCs) [51]. Bevacizumab and H-1PV therefore converge on the possession of several immune system boosting effects, which is likely to be the reason behind the favorable response obtained in glioblastoma patients subjected to combined treatment with both agents.

Following the successful completion of ParvOryx01, a second H-1PV clinical trial (ParvOryx02) was launched, which aimed to assess virus combination with gemcitabine for the treatment of inoperable metastatic pancreatic cancer [52]. The immunomodulating properties of gemcitabine have not yet been clearly documented. Nonetheless, Suzuki et al. [53,54] reported that this chemotherapeutic drug selectively eliminates splenic MDSCs and exerts significant immune stimulation in murine tumor models. In the ParvOryx02 trial setting, H-1PV oncolytic and immunomodulating capacities are expected to synergize with the cytotoxic (and immunostimulatory?) effects of gemcitabine. ParvOryx02 outcome evaluation is currently ongoing and will provide further experience of value in the development of H-1PV-based cancer viro-immunotherapies.
